# PSCA-CAR T cell therapy in metastatic castration-resistant prostate cancer: a phase 1 trial

**DOI:** 10.1038/s41591-024-02979-8

**Published:** 2024-06-12

**Authors:** Tanya B. Dorff, M. Suzette Blanchard, Lauren N. Adkins, Laura Luebbert, Neena Leggett, Stephanie N. Shishido, Alan Macias, Marissa M. Del Real, Gaurav Dhapola, Colt Egelston, John P. Murad, Reginaldo Rosa, Jinny Paul, Ammar Chaudhry, Hripsime Martirosyan, Ethan Gerdts, Jamie R. Wagner, Tracey Stiller, Dileshni Tilakawardane, Sumanta Pal, Catalina Martinez, Robert E. Reiter, Lihua E. Budde, Massimo D’Apuzzo, Peter Kuhn, Lior Pachter, Stephen J. Forman, Saul J. Priceman

**Affiliations:** 1 Department of Medical Oncology and Therapeutics Research, City of Hope, Duarte, CA USA; 2 Department of Computational and Quantitative Medicine, City of Hope, Duarte, CA USA; 3 Department of Hematology and Hematopoietic Cell Transplantation, City of Hope, Duarte, CA USA; 4 Departments of Mathematics and Biology and Biological Engineering, California Institute of Technology, Pasadena, CA USA; 5 Michelson Center for Convergent Bioscience, Convergent Science Institute in Cancer, University of Southern California, Los Angeles, CA USA; 6 Department of Immuno-Oncology, Beckman Research Institute of City of Hope, Duarte, CA USA; 7 Department of Radiology, City of Hope, Duarte, CA USA; 8 Department of Clinical and Translational Project Development, City of Hope, Duarte, CA USA; 9 Department of Urology, Jonsson Comprehensive Cancer Center, University of California, Los Angeles, CA USA; 10 Department of Pathology, City of Hope, Duarte, CA USA

**Keywords:** Prostate cancer, Immunotherapy, Cell therapies

## Abstract

Despite recent therapeutic advances, metastatic castration-resistant prostate cancer (mCRPC) remains lethal. Chimeric antigen receptor (CAR) T cell therapies have demonstrated durable remissions in hematological malignancies. We report results from a phase 1, first-in-human study of prostate stem cell antigen (PSCA)-directed CAR T cells in men with mCRPC. The starting dose level (DL) was 100 million (M) CAR T cells without lymphodepletion (LD), followed by incorporation of LD. The primary end points were safety and dose-limiting toxicities (DLTs). No DLTs were observed at DL1, with a DLT of grade 3 cystitis encountered at DL2, resulting in addition of a new cohort using a reduced LD regimen + 100 M CAR T cells (DL3). No DLTs were observed in DL3. Cytokine release syndrome of grade 1 or 2 occurred in 5 of 14 treated patients. Prostate-specific antigen declines (>30%) occurred in 4 of 14 patients, as well as radiographic improvements. Dynamic changes indicating activation of peripheral blood endogenous and CAR T cell subsets, TCR repertoire diversity and changes in the tumor immune microenvironment were observed in a subset of patients. Limited persistence of CAR T cells was observed beyond 28 days post-infusion. These results support future clinical studies to optimize dosing and combination strategies to improve durable therapeutic outcomes. ClinicalTrials.gov identifier NCT03873805.

## Abstract (web summary)

A phase 1 trial of PSCA-targeting CAR T cell therapy in patients with PSCA-positive, metastatic castration-resistant prostate cancer demonstrates that the treatment, using a protocol with reduced lymphodepletion, is safe and shows preliminary clinical activity.

## Main

Metastatic castration-resistant prostate cancer (mCRPC) is a lethal disease, causing more than 30,000 deaths in American men each year^1^. Immunotherapy has largely been unsuccessful; both vaccine-based strategies such as GVAX and Prost-VAC^2,3^ and immune-checkpoint inhibition with CTLA-4 and PD-1 inhibitors^4,5^ have shown limited activity. The only immunotherapy proven to prolong survival in mCRPC is sipuleucel-T, which is an autologous cellular immunotherapy with ex vivo incubation of dendritic cells leading to activation against prostate acid phosphatase^6^; however, significant improvements are needed for immunotherapies to effectively target mCRPC.

Reasons for the lack of immunotherapy response in prostate cancer are multi-fold, including strong immunosuppression in advanced prostate cancer^7^ that limits both trafficking and effector T cell function in the local tumor microenvironment. Despite this, there are unique tumor-associated antigens in mCRPC that are commonly and robustly expressed, including prostate stem cell antigen (PSCA) and prostate-specific membrane antigen (PSMA), which could be leveraged as targets for powerful cellular immunotherapy modalities. The dramatic successes of chimeric antigen receptor (CAR) T cell therapies in hematological malignancies have inspired the clinical development of CAR T cell therapies for the treatment of mCRPC.

PSCA is highly expressed in prostate cancer and increases with advanced disease states, particularly in the setting of bone metastases^8^. Using xenograft and syngeneic tumor models, we demonstrated safety and efficacy of second-generation PSCA-CAR T cells with 4-1BB co-stimulation in eradicating bone metastatic prostate cancer^9,10^. Here, we report results of our first-in-human phase 1 clinical trial to evaluate the safety and bioactivity of PSCA-CAR T cells in patients with mCRPC.

## Results

### Clinical trial design and patient characteristics

City of Hope conducted a single-center, first-in-human, phase 1 clinical trial to evaluate safety and bioactivity of PSCA-directed CAR T cells in patients with mCRPC (NCT03873805). The primary end points were safety and dose-limiting toxicities (DLTs). The secondary end points were expansion and persistence of CAR T cells to 28 days post-infusion (defined as CAR T cell percentage of total CD3^+^ cells in peripheral blood by flow cytometry, or at least 7.5 copies per ug of DNA in peripheral blood by qPCR), disease response (prostate-specific antigen (PSA) decline and Response Evaluation Criteria in Solid Tumors (RECIST)) and survival described as percent of participants alive at 6 months. Exploratory end points were phenotypes and frequencies of immune cell subsets in the peripheral blood pre- and post-therapy, serum cytokine profile before and after CAR T infusion to assess potential cytokine release syndrome (CRS) toxicity and CAR T cell effector function, phenotype of tumor-infiltrating lymphocytes, gene expression (by RNA sequencing) of circulating tumor cells (CTCs), cell-free DNA (cfDNA) in peripheral blood by whole-exome sequencing and CAR immunogenicity (anti-PSCA-CAR antibodies). The gene expression in CTCs, cfDNA in peripheral blood and CAR immunogenicity are not presented in this report.

**Fig. 1:**
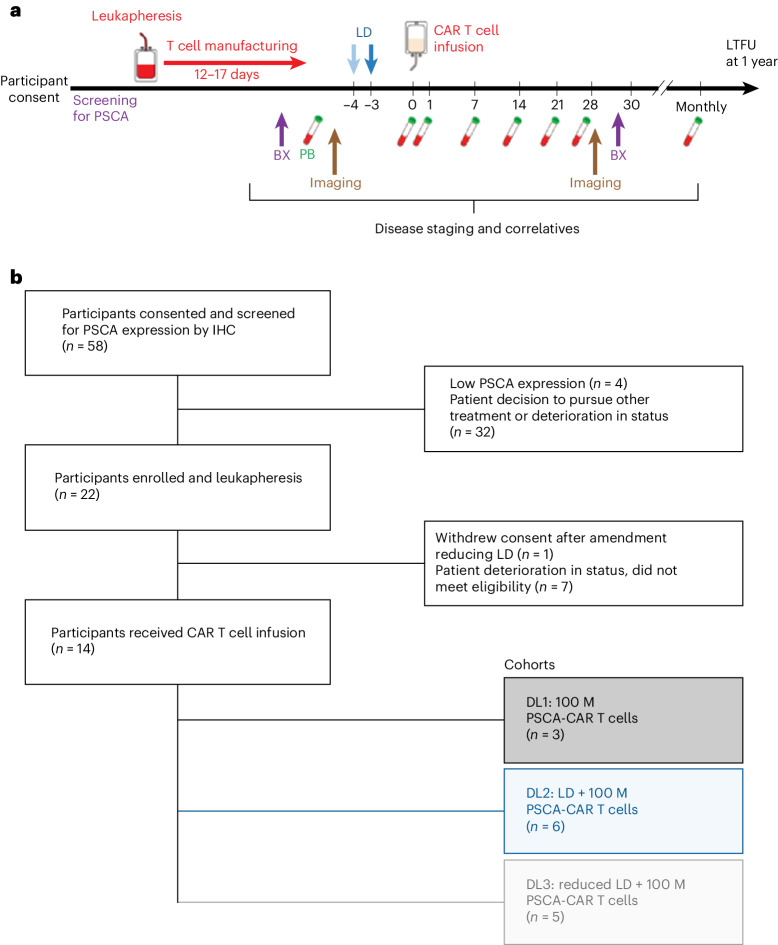
Clinical trial design and CONSORT diagram. **a**, Illustration of clinical trial design, including participant screening, leukapheresis, PSCA-CAR T cell manufacturing, pre-infusion biopsy (BX), peripheral blood (PB) sample collection before LD, bone scan and CT imaging, Flu/Cy LD, PSCA-CAR T cell infusion, serial PB sample collection time points from day 0 to day 28, post-infusion bone scan and CT imaging, post-infusion BX and long-term follow up (LTFU). **b**, CONSORT diagram detailing participants consented and screened for PSCA expression by immunohistochemistry (IHC) (*n* = 58), participants enrolled and leukapheresis (*n* = 22), participants received CAR T cell infusion (*n* = 14). DL cohorts, including DL1 (100 M PSCA-CAR T cells, *n* = 3), DL2 (Flu/Cy LD + 100 M PSCA-CAR T cells, *n* = 6) and DL3 (reduced Flu/Cy LD + 100 M PSCA-CAR T cells, *n* = 5). CT, computed tomography. Source data

The clinical trial design is summarized in Fig. 1a. Fifty-eight participants were screened for PSCA expression by immunohistochemistry (Extended Data Table 1 details the PSCA scores), 22 participants underwent leukapheresis and CAR T cell manufacturing and 14 participants were treated from August 2019 to July 2022 (Fig. 1b). The first participant was pre-screened (tissue testing) on 23 May 2019, the first participant was enrolled (leukapheresis) on 30 July 2019 and the last participant was treated (CAR T cell infusion) on 25 July 2022; the trial was then closed. Clinical and disease characteristics of the 14 participants are listed in Table 1. The median age of participants in the study was 62 years for dose level (DL)1, 70 years for DL2 and 69 years for DL3. All participants received previous androgen receptor signaling inhibitors, either enzalutamide (71%), abiraterone (79%) or both (64%), and a majority of patients received cabazitaxel (57%), docetaxel (86%) or both (57%) before CAR T cell infusion. Baseline PSA (median) ranged from 16.5 to 235.3.

**Table 1:** Patient characteristics

	DL1: 100 M CAR T cells, *n* = 3	DL2: LD + 100 M CAR T cells, *n* = 6	DL3: reduced LD + 100 M CAR T cells, *n* = 5
Age (years) median (range)	62 (59–69)	70 (42–73)	69 (62–72)
Race:			
White	3 (100)	6 (100)	3 (60)
Black	0	0	2 (40)
Asian	0	0	0
Other/unknown	0	0	0
Previous treatment:			
Enzalutamide	3 (100)	5 (83)	2 (40)
Abiraterone	3 (100)	6 (100)	2 (40)
Both	3 (100)	5 (83)	1 (20)
Previous treatment:			
Docetaxel	2 (67)	5 (83)	5 (100)
Cabazitaxel	2 (67)	3 (50)	3 (60)
Both	2 (67)	3 (50)	3 (60)
Baseline PSA median (range)	16.5 (10.7–20.4)	88.0 (11.7–590.2)	235.3 (1.79–3,260)
Lymph node only	2 (67)	1 (17)	0
Bone ± lymph node	0	4 (67)	4 (80)
Visceral	1 (33)	1 (17)	1 (20)

### CAR T cell product manufacturing and characterization

**Extended Data Fig. 1:**
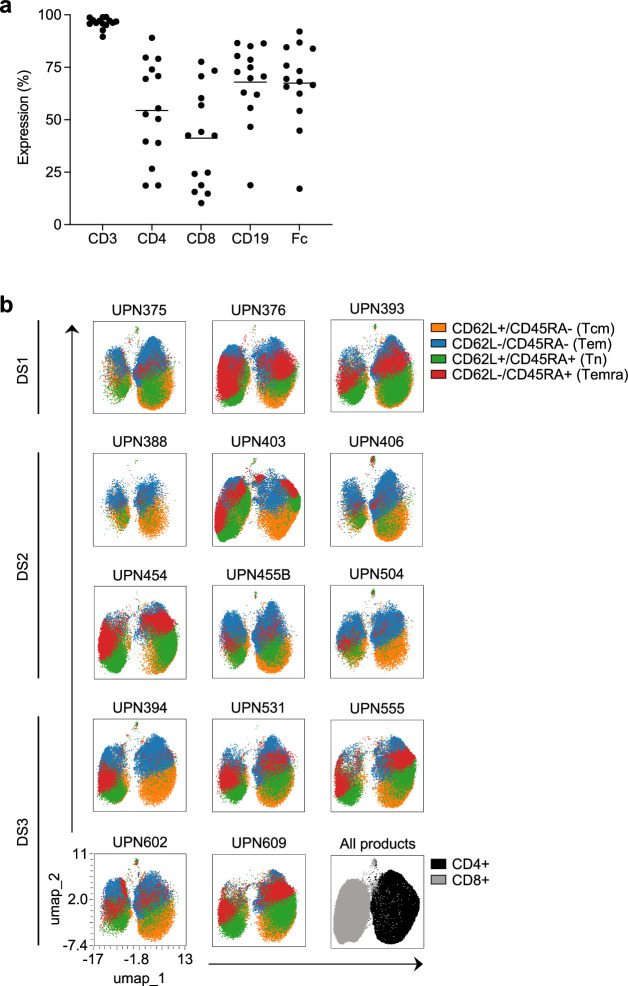
PSCA-CAR T cell product characterization. **(a)** Flow cytometric analysis of expression of CD3, CD4, CD8, CD19 (CD19t transduction marker), Fc (extracellular spacer domain in CAR) in CAR T cell manufactured products (individual dots are a single patient product). **(b)** Analysis of T cell subsets (Tn: CD62L + /CD45RA+ naïve, Tcm: CD62L + /CD45RA- central memory, Tem: CD62L-/CD45RA- effector memory, and Temra: CD62L-/CD45RA+ terminally differentiated effector memory) in CAR T cell manufactured products. Data are from n = 14. Source data

The PSCA-CAR construct comprised the anti-PSCA humanized scFv (A11 clone), ΔCH2 extracellular spacer, CD4 transmembrane domain, 4-1BB intracellular co-stimulatory domain and CD3γ cytolytic domain as previously published (Supplementary Fig. 1)^9^. In brief, CAR T cell manufacturing included depletion of CD14^+^ and CD25^+^ cells, CD3/28 bead stimulation, transduction with lentivirus at multiplicity of infection of 0.3, removal of beads at day 7–9, followed by expansion for a total of 12–17 days in interleukin (IL)-2 and IL-15 cytokines. There were no manufacturing failures, with a median CAR percentage of 86.8% in the final released product. Thawed products were characterized by flow cytometry for expression of CD4/CD8, CD19 (for CD19t transduction marker) expression and Fc (PSCA-CAR) expression (Extended Data Fig. 1a), as well as T cell subsets demonstrating a dominant T_CM_/T_EM_ phenotype (Extended Data Fig. 1b). Two products fell outside the prespecified woodchuck post-transcriptional regulatory element (WPRE) copy number (<5) and US Food and Drug Administration (FDA) approval was granted to proceed with the infusion. The median time from leukapheresis to infusion of the product was 73 days (range 34–182); delays were primarily due to protocol-mandated holds on accrual during toxicity assessments and protocol amendments, waiting for confirmatory PSCA staining from on-study biopsies, as well as seeking regulatory approval for the use of the product out of parameters (as specified above). Six patients received bridging therapy: cabazitaxel (*n* = 4), cabazitaxel + carboplatin (*n* = 1) and enzalutamide (*n* = 1).

### Treatment response

**Fig. 2:**
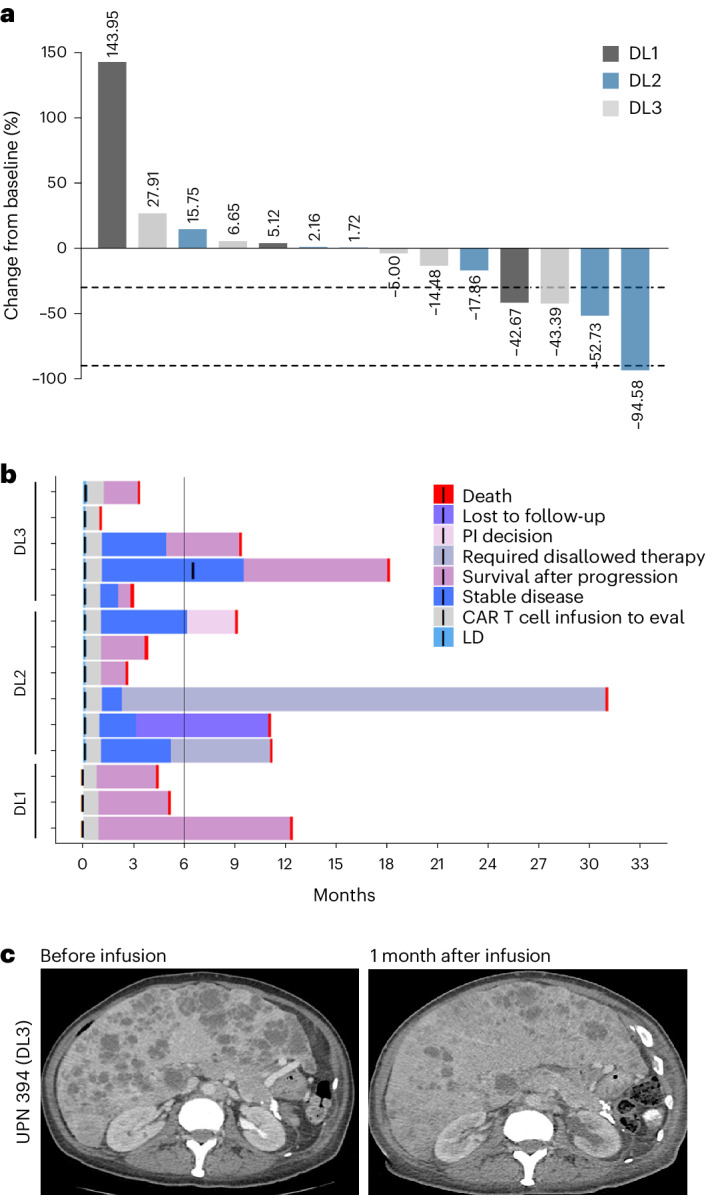
Treatment response following PSCA-CAR T cell infusion. **a**, PSA waterfall plot showing best PSA response in the 28 days following CAR T cell infusion at each DL. **b**, Swimmer’s plot depicting response to treatment and follow up for each participant on study. PI, principal investigator. **c**, CT scan of a patient (UPN394) in DL3 showing liver metastases before infusion and disease response 1 month after infusion of PSCA-CAR T cells. Source data

Declines in PSA levels from before treatment to day 28 after CAR T cell infusion were seen in 1 of 3 participants in DL1, 3 of 6 participants in DL2 and 3 of 5 participants in DL3 (Extended Data Table 2). A waterfall plot of the maximum PSA change from before CAR T cell infusion to day 28 shows 4 of 14 participants with PSA declines >30% (Fig. 2a). Of these, only one individual maintained a PSA decline >30% beyond 28 days. In DL1, one of three participants treated experienced a transient PSA response; notably this individual had evidence of early neuroendocrine (NE) expression in the on-study biopsy but still retained strong PSCA expression and the RECIST response was progressive disease. Post-treatment biopsy revealed further NE transformation (data not shown). The first participant treated in DL2 (with lymphodepletion) achieved a >90% PSA decline in the first 28 days post-CAR T cell infusion. The response in this individual is characterized in greater detail below.

RECIST best objective responses are displayed in Extended Data Table 2; rates of stable disease by RECIST were 0% (DL1), 67% (DL2) and 60% (DL3). Swimmer plots for treated participants are shown in Fig. 2b, with a 33%, 67% and 40% 6-month survival rate in DL1, DL2 and DL3, respectively (Extended Data Table 2). The first participant treated in DL3 achieved radiographic improvement in liver metastatic burden, but did not achieve a PSA response (Fig. 2c). One individual with bone only disease who exhibited stable disease in DL3 requested and received a second infusion of 100 million (M) CAR T cells about 6 months following initial infusion. He experienced transient relief of cancer-related pain after the second infusion.

We also evaluated treatment response by CTC quantification in the peripheral blood of treated participants on study using high-definition single-cell analysis (HDSCA)^11–13^. Cytokeratin (CK)-positive cells were detected in the peripheral blood of 100% of treated individuals. The median and mean changes in CTCs are depicted in Supplementary Table 1. Overall, there were marked declines in mean CK^+^ cells from baseline to 28 days after CAR T cell infusion in both lymphodepletion (LD) cohorts (DL2 and DL3), but not in DL1.

Somatic DNA sequencing results were available for eight individuals and two individuals had germline testing results (no overlap between somatic and germline-tested patients). Of the eight with somatic testing, the highest tumor mutational burden was 10.5, all others were deemed low. PTEN loss was noted in three of these individuals, one of whom experienced the greatest PSA decline on study (Fig. 2a). In DL3, the individual with radiographic improvement in liver metastases had a genomic alteration in CDK12 and he had progressed on previous immune-checkpoint inhibitor therapy.

### CAR T cell persistence and bioactivity

**Fig. 3:**
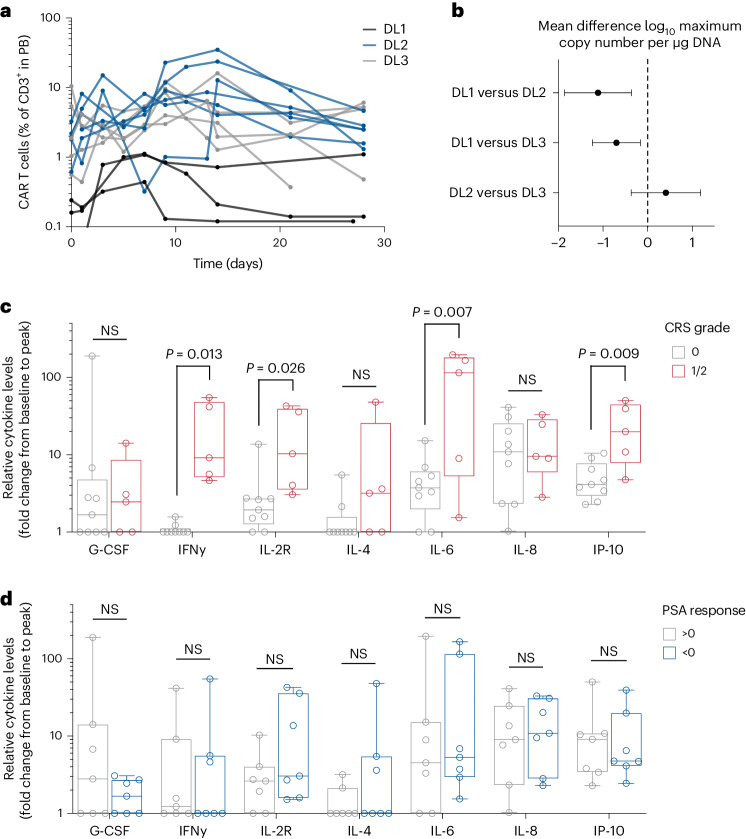
CAR T cell kinetics and serum cytokine analysis. **a**, Flow cytometric analysis of PSCA-CAR T cells (%) within CD3^+^ T cells in the PB for each individual. **b**, PSCA-CAR T cells in PB detected by quantitative PCR of WPRE copies per μg DNA. Data shown are 95% confidence limits for the mean differences of the three DLs in log_10_ maximum copy number per μg of DNA. **c**, Box plots of relative cytokine levels for each participant grouped by CRS grade 0 or 1/2. **d**, Box plots of relative cytokine levels for each individual grouped by PSA response <0 or >0. Data are from *n* = 14 and presented as mean values ± s.e.m. *P* values indicate differences between groups using a two-tailed Student’s *t*-test. NS, not significant. Source data

CAR T cell persistence/expansion kinetics in peripheral blood was assessed by flow cytometry of CD3^+^ T cells coexpressing the CD19t lentiviral transduction marker. CAR T cell expansion was enhanced by fludarabine/cyclophosphamide (Flu/Cy) LD (DL2 and DL3 versus DL1) and not significantly impaired with the reduced LD regimen in DL3 compared to DL2 (Fig. 3a and Supplementary Fig. 2a) acknowledging that the sample size is too limited to establish equivalence of DL2 and DL3. Increased expression of the exhaustion/activation marker PD-1, activation marker CD25 and memory antigen CD95 among CAR T cells was observed in peripheral blood in DL2 and DL3, which was greater than in DL1 (Supplementary Fig. 2b–d). We confirmed CAR T cell kinetics in peripheral blood by quantitative PCR for the presence of the WPRE within the lentiviral cassette, showing increased copies per μg DNA in individuals in the Flu/Cy LD cohorts (DL2 and DL3) compared to those without LD (DL1), which were not different from DL2 and DL3 (Fig. 3b). Despite the minimal observed differences between DL2 and DL3 in terms of expansion of CAR T cells in peripheral blood (Fig. 3a), reduced LD was associated with reduced CRS-related and off-tumor toxicities (detailed below).

### Toxicity assessment

Rates of DLT and CRS events are detailed in Supplementary Table 2, with two DLTs in DL2, and a maximum grade 2 CRS among the 14 participants on study. There were no DLTs in the first three participants treated at DL1 (without LD). In DL2, there was one DLT (non-infectious cystitis), resulting in enrollment of three additional participants at this DL. A second DLT cystitis event was observed in the next three participants. The cystoscopic appearance of cystitis was not distinctive. Intravesical therapy was not particularly effective and systemic steroid therapy was required in both DLT cases, after which the protocol management team met and amended the protocol to attempt to reduce the toxicity risk. Changes at DL3 included reducing the dose of LD chemotherapy cyclophosphamide from 500 to 300 mg m^−2^, with mandatory mesna, more frequent monitoring of urinalysis and enhanced guidance on management of cystitis. DL3 received the same dose of 100 M CAR T cells with these modifications and no DLT was seen in five participants; the maximum grade of cystitis was grade 2.

**Extended Data Fig. 2:**
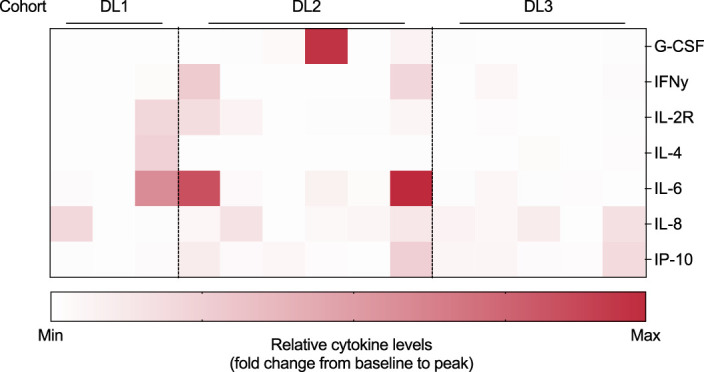
Serum cytokine analysis. Heat map showing relative fold change from baseline to peak cytokine levels of G-CSF, IFNy, IL-2R, IL-4, IL-6, IL-8, and IP-10 in each subject on study. Source data

CRS occurred in 5 of 14 participants (36%), 1 in DL1 (33%), 2 in DL2 (33%) and 2 in DL3 (40%) (Supplementary Table 2). The median onset of CRS was 4 days (range 3–8 days). Tocilizumab was administered in three individuals to mitigate symptomatic fever, although none of the CRS events reached grade 3 severity. No high-grade CAR T cell related neurologic toxicities or macrophage activation syndrome events were noted. Grade ≥3 neutropenia was noted in 0 of 3 (0%) at DL1, 6 of 6 (100%) individuals at DL2 and 3 of 5 (60%) at DL3. Attributed adverse events by category, MedDRA code and DL are summarized in Table 2. Induction of serum cytokines was significantly associated with clinical CRS grade (Fig. 3c and Extended Data Fig. 2). Serum cytokine induction was not significantly associated with PSA response (Fig. 3d).

**Table 2:** Adverse events^a^

Arm	Category	MedDRA code	Grade	
			2	3
DL1, *n* = 3	Blood and lymphatic system disorders	Anemia	0	2
	Investigations	Lymphocyte count decreased	0	1
	Metabolism and nutrition disorders	Hypoalbuminemia	2	0
		Hyponatremia	2	0
	Skin and subcutaneous tissue disorders	Rash maculo-papular	2	0
DL2, *n* = 6	Blood and lymphatic system disorders	Anemia	1	2
	General disorders and administration site conditions	Fatigue	2	2
		Fever	2	0
		Pain	0	1
	Metabolism and nutrition disorders	Anorexia	2	0
	Renal and urinary disorders	Bladder spasm	2	0
		Cystitis noninfective	1	2
		Hematuria	2	1
		Proteinuria	2	0
	Skin and subcutaneous tissue disorders	Rash maculo-papular	1	1
DL3^b^, *n* = 5	Investigations	Lymphocyte count decreased	0	1

^a^All grade 3 events and grade 2 events that occurred in more than one participant.

^b^One participant received only cyclophosphamide, without fludarabine, due to a shortage.

### Patient with biochemical and radiographic response

**Fig. 4:**
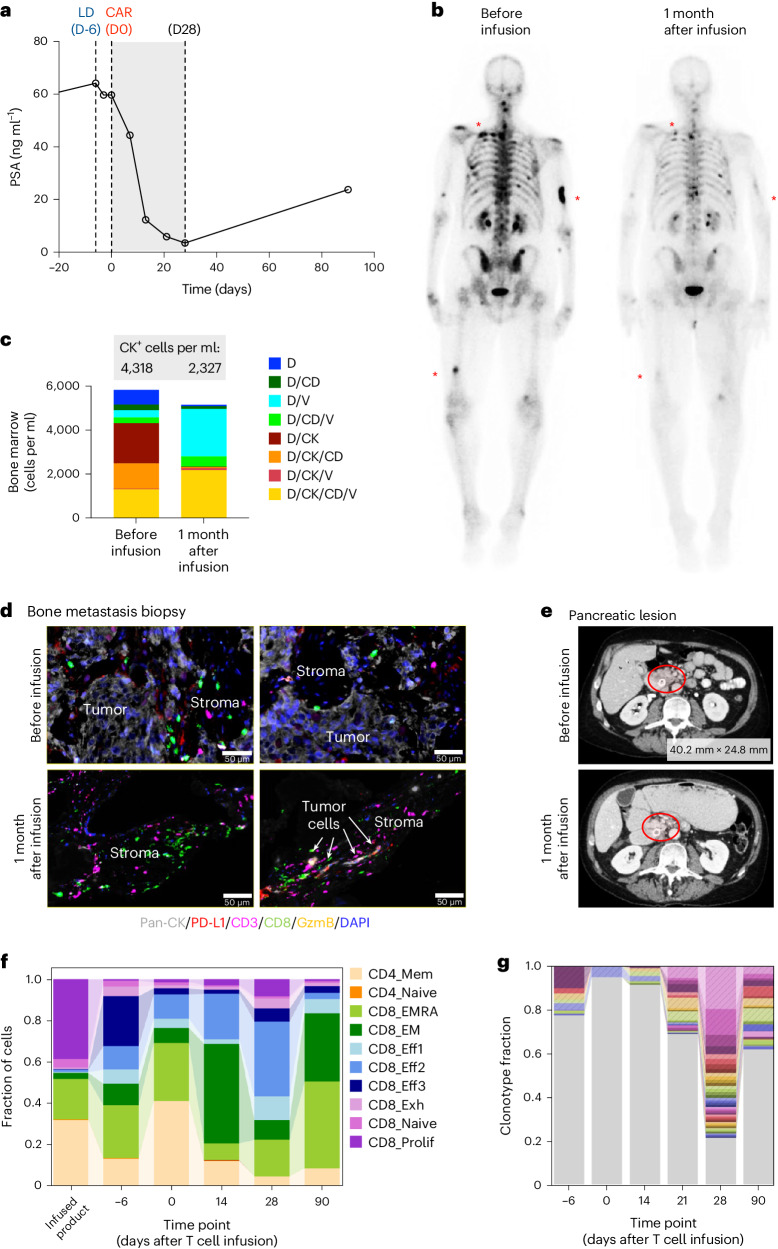
Patient with biochemical and radiographic response with associated immune landscape changes. **a**, PSA response in UPN388 on DL2 before and through the 28 days following PSCA-CAR T cell infusion and at day 90. **b**, Bone scintigraphy (anterior–posterior view) for bone metastases detection before and 1 month after PSCA-CAR T cell infusion in the same patient. Red asterisks denote representative bone metastases. **c**, HDSCA of CTCs in the bone marrow before and 1 month after infusion of PSCA-CAR T cells. Quantification of CK^+^ cells per ml is shown in the gray box. **d**, Immunofluorescence images of bone metastasis biopsy samples from before (top) and 1 month after PSCA-CAR T cell infusion (bottom), evaluating expression of pan-CK (tumor cells), PD-L1, CD3 (T cells), CD8 (effector cells) and Granzyme B (GzmB). Indicated areas of tumor and stromal regions and arrows indicate residual tumor cells in post-infusion sample. Images shown are representative of the whole evaluable tissue region on slide. DAPI, 4,6-diamidino-2-phenylindole. **e**, CT scan of pancreatic lesion in UPN388 before and 1 month after PSCA-CAR T cell infusion. Red circles denote pancreatic lesion around stent. Measured size of lesion before infusion, 40.2 × 24.8 mm. The lesion regressed 1 month after infusion and was not measurable. **f**, scRNA-seq analysis of CD3^+^ T cell subsets in the infused product and in PB T cells at the indicated time points after T cell infusion. **g**, Single-cell analysis of TCRα/β repertoire diversity in PB T cells at the indicated time points after T cell infusion. Top 40 clonotypes with the greatest fractions at day 28. Source data

**Extended Data Fig. 3:**
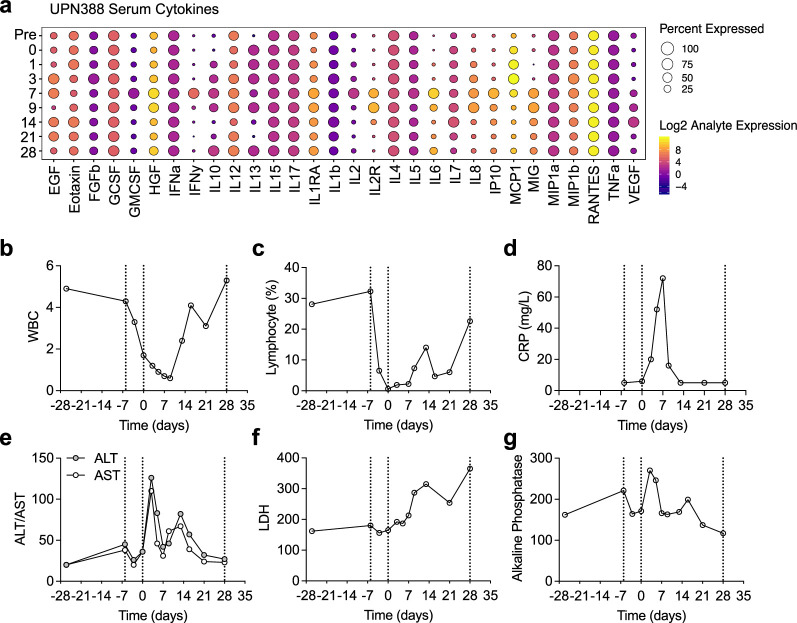
Serum cytokines and chemistry in UPN388. **(a)** Serum cytokine analysis for UPN388. **(b-g)** Quantification of white blood cells (WBCs) **(b)**, lymphocytes (%) **(c)**, C-reactive protein (CRP) **(d)**, ALT/AST **(e)**, lactate dehydrogenase (LDH) **(f)**, and alkaline phosphatase **(g)** in serum for UPN388. Source data

**Extended Data Fig. 4:**
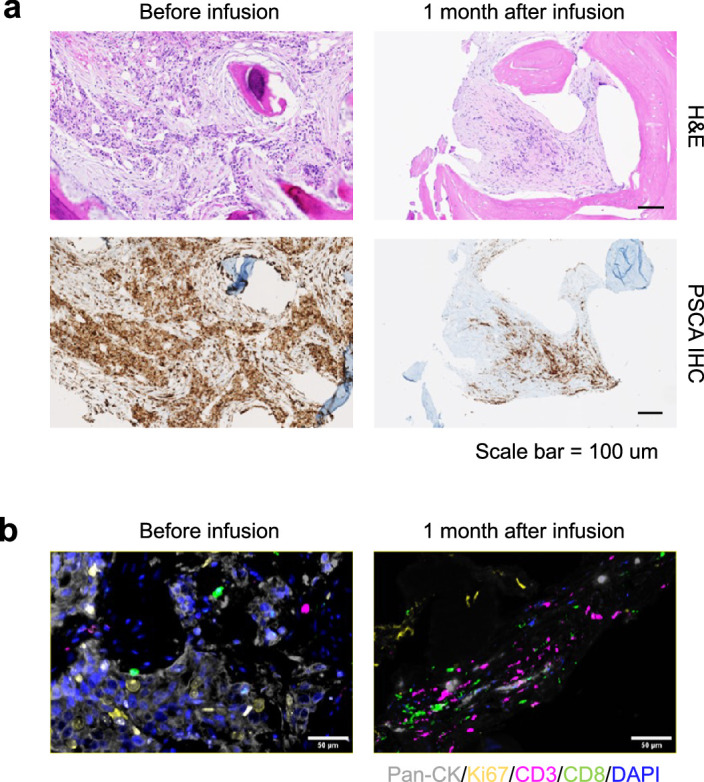
Analysis of UPN388 bone metastasis biopsy. **(a)** H&E (top) and PSCA IHC (bottom), and **(b)** immunofluorescence imaging before infusion and 1 month after infusion of PSCA-CAR T cells. Images shown are representative of the whole evaluable tissue region on slide. Source data

**Extended Data Fig. 5:**
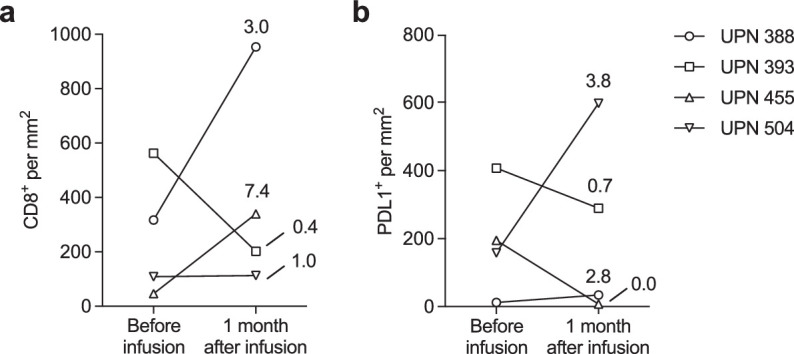
T cell infiltration and PD-L1 expression in biopsies. Quantification of CD8+ **(a)** and PD-L1+ **(b)** areas in biopsies by immunofluorescence imaging. Data shown includes all evaluable tissue on slide. Source data

One participant in DL2 experienced a >90% PSA decline following CAR T cell infusion, from 64.2 ng ml^−1^ before LD and CAR T cells to 3.5 ng ml^−1^ at day 28 after CAR T cell infusion (Fig. 4a). Radiographic improvement was seen in this individual’s soft tissue metastasis (Fig. 4b) although the RECIST assessment was ‘stable disease’ due to the presence of bone metastases. Changes in serum cytokines in this individual demonstrate pronounced but transient induction of inflammatory factors, including interferon (IFN)γ, IL-6, granulocyte–macrophage colony-stimulating factor (GM-CSF), IP-10 and MIG (Extended Data Fig. 3a). Serum chemistry showed mild increases in C-reactive protein (max 81 mg l^−1^), ferritin (max 555 ng ml^−1^), alanine transaminase (ALT)/aspartate transaminase (AST) (<1.5 × upper limit of normal (ULN)), lactate dehydrogenase (max 365 U l^−1^) and alkaline phosphatase (max 192) (Extended Data Fig. 3b–g) following CAR T cell infusion. This corresponded to grade 2 CRS with a *T*_max_ of 39.1 on day 4, 38.8 on day 5 and tocilizumab was administered on day 6 due to persistent rigors without fever; all aforementioned labs subsequently trended down by day 21. CTC assessment with CK positivity was significantly reduced, both in bone marrow (Fig. 4c) and in peripheral blood (Supplementary Fig. 3) samples, from baseline to 28 days after CAR T infusion. The post-CAR T cell infusion bone metastasis biopsy showed reductions in PSCA^+^ disease (Extended Data Fig. 4a) and Ki-67^+^ expression (Extended Data Fig. 4b), along with greater infiltration of CD3^+^ and cytotoxic CD8^+^ T cells by immunofluorescence staining (Fig. 4d). Few residual tumor cells in the post-treatment biopsy were observed and were associated with increased granzyme B^+^ and PD-L1^+^ areas, suggestive of an active antitumor immune response. Quantification of immunofluorescence staining showed increased CD8^+^ and PD-L1^+^ areas in this individual, with variable results from other individuals analyzed (Extended Data Fig. 5a,b). Notably, UPN388 also had a biopsy-proven prostate cancer metastasis in the pancreas that necessitated stent placement before study entry; this completely resolved after CAR T cell infusion (Fig. 4e).

### Immune landscape changes in patient with biochemical response

**Extended Data Fig. 6:**
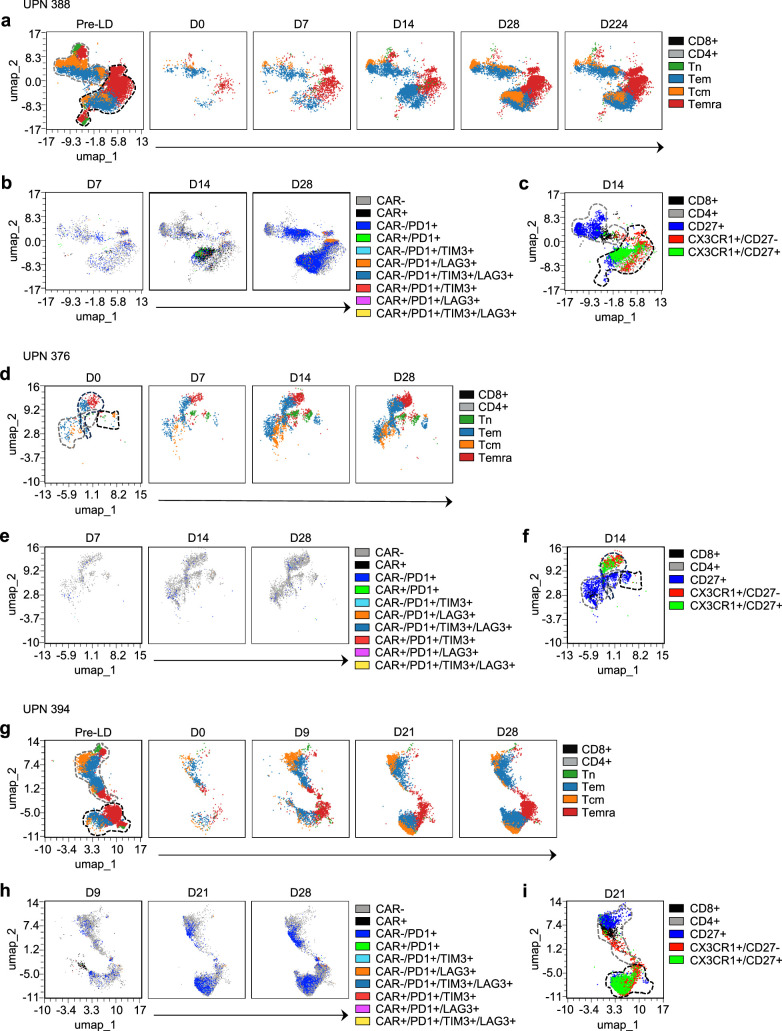
Flow cytometric analysis of peripheral blood CD8 + PD-1 + CAR and non-CAR T cells in UPN388, UPN375, and UPN394. **(a)** Flow cytometric analysis of peripheral blood T cells pre- and post-CAR T cell infusion for UPN388. Populations are categorized as CD8+, CD4+, and T cell subsets (Tn: CD62L + /CD45RA+ naïve, Tcm: CD62L + /CD45RA- central memory, Tem: CD62L-/CD45RA- effector memory, and Temra: CD62L-/CD45RA+ terminally differentiated effector memory). **(b)** Expression of PD-1, LAG3, and TIM3 on non-CAR (CAR-) and CAR + T cells at indicated time points. **(c)** Expression of CX3CR1 and CD27 at day 14 post-CAR T cell infusion. **(d-f)** Same as (a-c) for UPN375 (DL1). **(g-i)** Same as (a-c) for UPN394 (DL3). Source data

**Extended Data Fig. 7:**
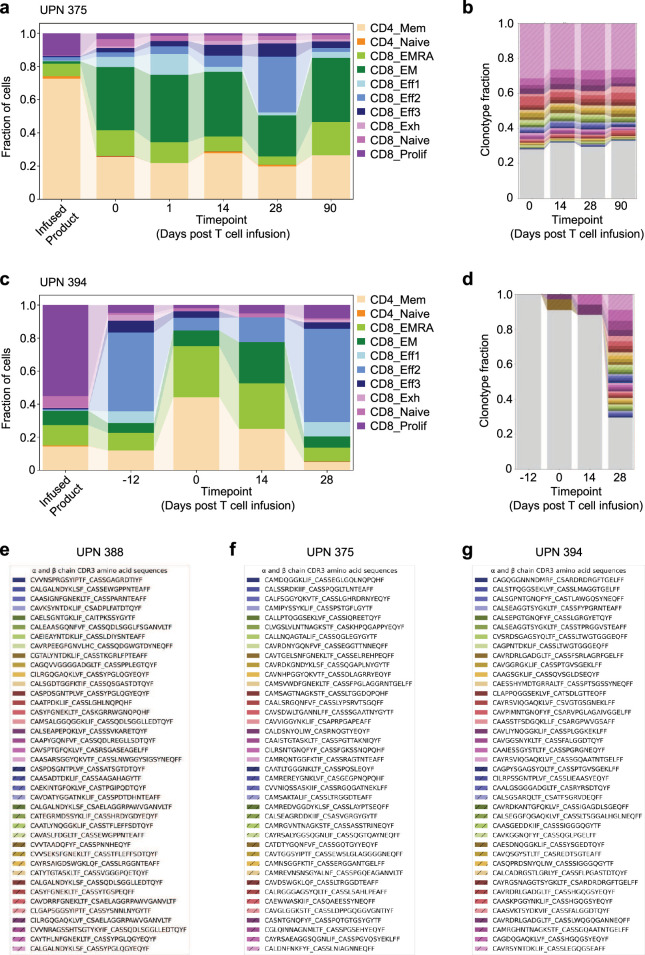
Single-cell analysis of CD3 + T cell subsets and TCRα/β repertoire diversity in the peripheral blood. **(a)** scRNA-seq analysis of CD3 + T cell subsets in the infused product and in the peripheral blood T cells at indicated time points post-T cell infusion of UPN375. **(b)** Single-cell analysis of TCRα/β repertoire diversity in the peripheral blood T cells of UPN375 at indicated time points post-T cell infusion. **(c-d)** Same as (a-b) for UPN394. **(e)** Legend for Fig. 4g. **(f)** Legend for Extended Data Fig. 7b. **(g)** Legend for Extended Data Fig. 7d. Source data

**Extended Data Fig. 8:**
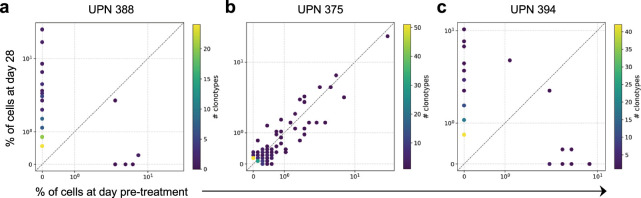
TCR repertoire diversity in peripheral blood T cells. Number of cells per clonotype at day 0 versus day 28 following therapy in UPN388 (**a)**, UPN375 (**b)**, and UPN394 **(c)**. Source data

Endogenous and CAR T cell populations in peripheral blood were further characterized by flow cytometry, as well as by single-cell RNA sequencing (scRNA-seq) and TCR repertoire analysis in this patient. Initial assessment of T cell subsets in peripheral blood pre- and post-LD and CAR T cell infusion in this patient showed dynamic changes in naive (T_N_), central memory (T_CM_), effector memory (T_EM_) and terminally differentiated effector memory (T_EMRA_) cells over time (Extended Data Fig. 6a). Greater re-emergence of CD8^+^ T_CM_ and T_EM_ cells were observed by day 28 post-CAR T cell infusion. CD8^+^ CAR T cells expanded with this phenotype by day 14 in this patient (Extended Data Fig. 6a). Notably, CAR^+^ and endogenous non-CAR T cells showed increased PD-1 expression (and smaller increases in LAG3 and TIM3) over the 28 days following treatment (Extended Data Fig. 6b and Supplementary Fig. 4), which is associated with an activation and/or exhaustive phenotype. Peripheral blood CAR T cells showed elevated expression of CX3CR1 (Extended Data Fig. 6c), which has been correlated with response to immunotherapy with anti-PD-1 immune-checkpoint blockade^14^. Few CX3CR1-positive T cells were observed in the product before infusion. Similar results in CAR^+^ and endogenous non-CAR T cells in the peripheral blood were observed in a patient in DL3 but not in DL1 (Extended Data Fig. 6d–i). scRNA-seq corroborated these data, with increased effector CD8^+^ T cell subsets, including CX3CR1^+^CD8^+^ T cells in patients (Fig. 4f and Extended Data Fig. 7a,c). Single-cell TCRa/b repertoire analysis of endogenous T cells in peripheral blood demonstrated emerging and expanded clones by day 28 post-CAR T cell infusion in patients (Fig. 4g and Extended Data Figs. 7b,d,e–g and 8), which contracted at days 90 in UPN388, suggesting TCR clonal diversity changes following therapy. Collectively, these data suggest that LD + PSCA-CAR T cell therapy can induce biochemical and radiographic response along with changes in the immune landscape and TCR repertoire.

## Discussion

CAR T cell therapy has achieved durable response rates for patients with refractory hematological malignancies^15–17^, creating enthusiasm in translating this therapy to patients with solid tumors. Our study evaluated PSCA-directed CAR T cells in patients with mCRPC. We observed biochemical and radiographic responses in patients following LD and PSCA-CAR T cell infusion. The DLT was cystitis, which was likely an on-target/off-tumor effect^18^ with contribution from cyclophosphamide LD^19^. Reducing the cyclophosphamide dose avoided high-grade cystitis events in DL3 while retaining similar peripheral blood expansion of CAR T cells, although the small number of patients limits the statistical power to exclude a difference. In this heavily pretreated population, encouraging anticancer responses were seen. Our findings are limited by the small number of participants accrued. Accrual to a phase 1 trial with a tissue pre-screening requirement holds to accrual during DLT assessment periods and enrollment of heavily pretreated patients was by nature slow, and many patients did not proceed with treatment if disease progression occurred in a way that led to ineligibility, including some who had undergone leukapheresis. The relatively lengthy process may have excluded patients with more aggressive disease or borderline performance status. This highlights the importance of streamlining enrollment in phase 2 to reduce enrollment bias and overall study cost by improving the rate of infusion of manufactured CAR T cell product. This study validates PSCA as a viable CAR T cell therapeutic target and provides encouraging early clinical data to support further studies, focused on extending CAR T cell persistence, which, with the use of new dosing and/or combinatorial strategies, is hoped to lead to improved responses in patients.

Both the activity and toxicity of CAR T cells were impacted by the addition of LD, although the role of LD in facilitating CAR T cell activity in solid tumors is likely different than the role it plays in hematological malignancies. Preconditioning with LD promoted greater peripheral blood CAR T cell expansion and serum cytokine levels, which manifested in greater objective anticancer response in DL2 and DL3. These phase 1 trial results validate our recent preclinical studies, which found that increased efficacy of CAR T cells following administration of cyclophosphamide was associated with enhanced T cell infiltration into tumors, along with antigen-presenting cell (dendritic cell) infiltration and reduced myeloid suppressive features compared to CAR T cell therapy alone^10^. Notably, lower-dose cyclophosphamide (300 mg m^−2^) still yielded greater CAR T cell bioactivity than the absence of LD, while it did reduce the toxicities compared to cyclophosphamide dosed at 500 mg m^−2^; similar findings have been documented in hematological malignancies^20^. Given the critical role of LD, an important avenue of investigation will be to study different LD regimens for optimal changes in the tumor immune microenvironment. Preclinically, Gene Ontology enrichment analysis identified T cell migration and IFNγ production as key processes enhanced by cyclophosphamide pretreatment^10^ and these can be used as end points of preclinical exploration. Metronomic dosing strategies of cyclophosphamide and alternative LD regimens warrant evaluation as the traditional high dose intravenous (i.v.) 3-day LD regimen adopted from hematological malignancy CAR T cell trials may not be equivalently translated for solid tumors. Taxanes and platinum agents have shown potential for modulation of the tumor immune microenvironment and for solid tumor CAR T cell therapy^21,22^ and bendamustine is also emerging as a potentially valuable LD agent^20^.

DLTs were only seen after the addition of LD in this trial, mirroring the toxicity experience of the PSMA-targeted TGFβ-dominant negative CAR T cell trial, in which DLTs were only encountered after LD chemotherapy was added^23^. In the PSMA-targeting TGFβ-insensitive armored CAR T cell trial, the dose of CAR T cells was reduced after high-grade toxicity occurred (sepsis and macrophage activation syndrome/hemophagocytic lymphohistiocytosis). Our approach of incorporating LD before CAR T cell dose escalation allowed for the earlier appearance of DLTs during the trial. Therefore, reducing LD and maintaining CAR T cell dose resulted in continued evidence of anticancer efficacy with a lower toxicity profile. CRS onset was slightly delayed with PSCA-CAR T cell therapy compared to the experience in hematological malignancies^24^, with a median onset at 4 days post-infusion in this study. Tocilizumab was administered in three patients primarily for relief of fever and chills, with no grade 3 CRS events and no hypotension or hypoxia events noted. Unlike other CAR T cell trials in mCRPC^23,25,26^, no high-grade neurologic toxicity nor macrophage activation syndrome/hemophagocytic lymphohistiocytosis events occurred, although it is unclear whether the PSCA target or this particular CAR T cell construct underlie this observation. Overall, the favorable toxicity profile of PSCA-CAR T cell therapy enables the currently accruing phase 1b trial (NCT05805371) to proceed with entirely outpatient dosing in the context of close clinical monitoring.

While PSCA-CAR T expansion was robust with objective measures of disease-modifying activity, including PSA decline, reduction in CTCs and radiographic improvements, there was a lack of CAR T cell persistence that corresponded to the lack of durable remission. Innovative strategies will be needed to enhance CAR T cell persistence and prolong the anticancer efficacy. Armoring CAR T cells with modifications such as the TGF-dominant negative approach with PSMA targeting showed efficacy but at the cost of fatal toxicities, perhaps due to uncontrolled over-expansion^23^. Over-expansion was also potentially the cause of fatal toxicity with the strategy employed by the Go-CAR T agent, in which rimiducid was administered in pulses to stimulate proliferation^26^. Alternative strategies to improve persistence of CAR T cells may include enriching for T naive/stem/memory cells^27^ and incorporating agents into the CAR T cell manufacturing process to improve T cell fitness, including AKT inhibitors^28,29^. Allogeneic cell therapy approaches may further modify therapeutic activity^30^ while also increasing feasibility by shortening time to treatment, which was a factor in the high drop-out rate observed in this trial. To avoid high-grade toxicity and to prolong the presence of infused T cells, our phase 1b strategy will administer multiple smaller doses of PSCA-CAR T cells rather than escalating to a larger single dose. Preclinical models of cystitis can be leveraged to study potential prevention or early intervention strategies, which could make it feasible to escalate the PSCA-CAR T cell dose in future trial iterations.

Tumor antigen heterogeneity with NE transformation was noted in one patient in this study, and may be a more general resistance mechanism in heavily treated patients with mCRPC. Treatment-emergent NE transformation is reported to occur more commonly in mCRPC recently since the introduction of powerful androgen receptor pathway inhibitors^31^ Thus, treating patients with mCRPC earlier in the disease course may be necessary to achieve durable responses. Alternatively, dual targeting to include proteins expressed on the de-differentiated CRPC cell populations such as CEA or DLL3 (ref. ^32^) may be required. T cell exhaustion may have contributed to the limited duration of activity, as indicated by upregulation of PD-1 in peripheral CAR T cells. Anecdotal experiences suggest that CAR T cells lose function in the setting of high tumor volumes, and immune-checkpoint inhibitor therapy may rescue incomplete CAR T cell responses^33^, which may have contributed to the long-term survival of a patient who received pembrolizumab as part of a clinical trial after participation in the phase 1 PSCA-CAR T study (Fig. 2).

In summary, our first-in-human phase 1 trial evaluating PSCA-CAR T cell therapy showed bioactivity and early evidence of clinical effectiveness, although on-target toxicity of cystitis impacted the intended CAR T cell dose escalation. Reduced LD dose mitigated toxicity while still enhancing CAR T cell expansion compared to no LD. Future studies will explore multi-dose and combinatorial strategies to improve persistence, with the goal of increasing clinical activity in patients with mCRPC.

## Methods

### Trial design and patients

This was a single-center phase 1 trial aimed at evaluating safety and feasibility of i.v. administered, lentivirally transduced PSCA-CAR T cells in patients with mCRPC, with a total of three DL cohorts. The primary end points were safety and DLTs. The secondary end points were persistence of CAR T cells to 28 days post-infusion (defined as CAR T cells comprising at least 7.5 copies per μg of DNA of total CD3 cells), expansion of CAR T cells (max log_10_ copies per μg of genomic DNA), disease response (PSA decline and RECIST) and survival described as percent of participants alive at 6 months. Exploratory end points were phenotypes and frequencies of immune cell subsets in the peripheral blood pre- and post-therapy, phenotype of tumor-infiltrating lymphocytes and serum cytokine profile before and after CAR T cell infusion to assess potential CRS toxicity and CAR T cell effector function. Additional planned exploratory end points not reported in this paper include gene expression (by RNA-seq) of CTCs, cfDNA in peripheral blood by whole-exome sequencing and CAR immunogenicity (anti-PSCA-CAR antibodies).

The trial was conducted in accordance with the US FDA and International Conference on Harmonization Guidelines for Good Clinical Practice, the Declaration of Helsinki and applicable institutional review board requirements (study protocol approved by the City of Hope Institutional Review Board). Only individuals with male sex were enrolled due to prostate cancer presenting only in this sex group. After a US FDA Investigational New Drug Application was obtained and the institutional review board approved the protocol, participants provided written informed consent in a two-step process. Many patients pre-screened (tissue PSCA testing) but did not proceed with leukapheresis due to lengthy wait times with limited slot availability and accrual pauses during DLT evaluation periods. The trial was registered with clinicaltrials.gov (NCT03873805). The City of Hope Data Safety Monitoring Board monitored the conduct of this study to ensure the safety of enrolled and treated participants and the validity and integrity of the acquired data.

A starting dose of 100 M PSCA-CAR T cells was selected based on experience with other CAR T cell trials and anticipated effective dose. The first three participants on DL1 at 100 M CAR T cells without Flu/Cy preconditioning LD and the first three participants on DL2 at 100 M CAR T cells with LD were staggered through the DLT period. All further participants were accrued to DLs in cohorts of three. After evaluation of the data from the completed DLT period (28 days) the protocol management team met to determine whether it was safe to escalate to the next DL, with rules following the toxicity equivalence range design of Blanchard and Longmate^34^ with an equivalence range of 0.20–0.35 and a too-toxic level of 0.51. The first cohort received 100 M CAR T cells without LD; the subsequent cohorts would all receive LD with plans to escalate the dose of CAR T cells from 100 M to 300 M to 600 M and the option to de-escalate the dose to 50 M if LD plus 100 M CAR T cells was not tolerated.

For LD chemotherapy, the standard regimen of cyclophosphamide 500 mg m^−2^ i.v. on days −5 to −3 and fludarabine 30 mg m^−2^ i.v. on days −5 to −3 was employed in DL2; this was reduced due to DLTs and DL3 participants received cyclophosphamide 300 mg m^−2^ i.v. on days −5 to −3 with the same dose schedule of fludarabine. Prophylactic G-CSF was not utilized, but G-CSF could be added for neutropenia if the treating physician felt it was indicated, as well as all other standard supportive measures such as antiemetics.

To attempt to exclude patients unlikely to benefit due to lack of tumor PSCA expression, all potential participants signed a pre-screening consent form so that archived tissue could be tested for PSCA by IHC staining. Participants were required to have at least moderate PSCA expression in their prostate primary or metastatic biopsy tissue to enroll in the study, although due to lack of a validated assay there was no prespecified cutoff. All participants enrolled had PSCA expression in ≥30% of tumor cells (Fig. 1a). An on-study biopsy was performed and for soft tissue metastases, confirmation of PSCA staining was required (this was not required for bone metastases due to inadequate calibration of the IHC assay on bone material); repeat biopsy of the same metastatic area was performed during the day 28 assessment period.

Patients with mCRPC were eligible if they had experienced disease progression on at least one androgen receptor pathway inhibitor (abiraterone or enzalutamide); previous taxane chemotherapy was allowed but not required. Creatinine clearance ≥50 ml min^−1^ was required, as well as AST/ALT ≤ 5 × ULN and bilirubin ≤ 2.0 mg dl^−1^. An electrocardiogram was required to show no acute abnormalities requiring intervention and an echocardiogram was required to document a left ventricular ejection fraction of ≥40%. Patients with clinically significant cardiac arrhythmias or central nervous system disease were excluded. Patients with HIV, active hepatitis B or C or uncontrolled active infection were excluded. Eligibility was confirmed before leukapheresis and again before the start of treatment (DL1, CAR T and DL2 or DL3, LD).

### CAR T cell manufacturing

Following screening and enrollment into the trial, participants underwent leukapheresis at City of Hope’s Michael Amini Transfusion Medicine Center. Autologous peripheral blood mononuclear cells were immunomagnetically depleted of CD14^+^ and CD25^+^ cells, then stimulated with CD3/CD28 DynaBeads and subjected to transduction with PSCA(dCH2)BBζ/CD19t lentivirus (multiplicity of infection of 0.3) followed by T cell expansion for 12–17 days until the freezing process. Cells were manufactured in the City of Hope Center for Biomedicine and Genetics GMP facility.

### Toxicity monitoring

Participants were hospitalized to receive study therapy and were required to stay in the hospital for at least 7 days after CAR T cell infusion. Additionally, they were required to have a full-time caregiver available and to stay within 40 min of the cancer center for the 28-day period following CAR T cell infusion. Toxicities were graded according to the Common Toxicity Criteria for Adverse Events v.4. DLTs were defined as any grade 3 or higher toxicity (grade 4 or higher hematologic toxicity), with the standard exceptions for CAR T cell protocols; and designated as definitely, probably or possibly related (level of attribution) to the infusion of the T cells and occurring within 28 days of T cell infusion. Specifically, any emergent grade 3 or greater organ toxicity (cardiac, pulmonary, gastrointestinal, hepatic or renal) with an attribution of possible, probable or definite to CAR T cells that are not pre-existing or due (at least possibly related) to underlying malignancy and lasting more than 72 h with intervention; any grade 3 or greater autoimmune toxicity and occurring within 28 days of T cell infusion; and any grade 5 toxicity with an attribution of possibly, probably or definitely related to the infusion of the T cells.

### Biological response assessment

PSA was measured at least every 2 weeks during the DLT period, then at days 60 and 90. Baseline imaging consisted of a conventional CT and bone scan. All imaging was repeated at days 28–30, then at day 90 and every 12 weeks thereafter, until progression of disease or the start of a new line of therapy.

### Flow cytometry

Peripheral blood samples were obtained from individuals before and at various time points for 28 days following CAR T cell infusion, as well as day 60, day 90 and q12 weeks after day 90 to evaluate CAR T cell expansion/persistence. Peripheral blood samples were lysed using BD PharmLyse (15 min at room temperature; RT) and quenched using RPMI containing 10% FBS. Cells were resuspended in FACS buffer (Hank’s balanced salt solution without Ca^2+^, Mg^2+^ or phenol red (HBSS^−/−^, Life Technologies) containing 2% FBS and 1 × AA). Cells were incubated with Fc block (BD Biosciences) for 5 min at RT and then incubated with fluorescence-labeled antibodies for 15 min at RT in the dark. Unless otherwise stated, antibodies were used at a dilution of 1:100. Supplementary Tables 3 and 4 provide full antibody lists and Supplementary Fig. 4 provides the flow cytometry gating strategy. Cell viability was determined using DAPI (Sigma, cat. no. D8417). For samples run on the Cytek Aurora, samples were thawed, counted using a Muse cell counter (1 M cells) and were stained in a two-step process. Before staining, the cells were Fc Blocked with BD Pharmingen Human BD Fc Block (BD Biosciences) for 20 min on ice, washed, spun and resuspended in the first master mix. The first master mix included one antibody, PD-L1 PE-Fire810 (BioLegend), in FACS buffer. Following incubation on ice for 20 min, cells were washed twice with FACS buffer and then stained with a 23-antibody master mix (Supplementary Table 4). The second master mix was prepared using FACS buffer with Brilliant Buffer Plus (BD Horizon). After incubation, the cells were washed twice with FACS buffer and finally resuspended in FACS buffer with 7-AAD (Invitrogen). Flow cytometry was performed on a MACSQuant Analyzer 10 (Miltenyi Biotec) or Cytek Aurora 3 and data were analyzed with FlowJo software (v.10.8.1, TreeStar) or OMIQ software (Dotmatics).

### WPRE quantitative PCR analysis

CAR T cell persistence in peripheral blood was determined by quantification of the WPRE region of the lentiviral transgene by qPCR. Genomic DNA was extracted from frozen whole blood samples using the QIAamp DNA Blood Mini kit according to the manufacturer’s protocol and WPRE copy number was measured by TaqMan qPCR. Average copy numbers were presented if at least two of three replicates generated a cycle threshold (Ct) value. Participants were measured for WPRE before and at the indicated time points following CAR T cell infusion.

### Serum cytokine analysis

Serum was collected and analyzed using the Human Cytokine 30-Plex Panel kit (Invitrogen) and Flexmap 3D (Luminex) according to the manufacturers’ protocols. Cytokine concentrations were calculated using Bio-Plex Manager v.6.0 software with a five parameter curve-fitting algorithm applied for standard curve calculations for duplicate samples.

### Single-cell transcriptomics and TCR repertoire analysis

Single-cell RNA and TCR libraries were prepared using 10x Genomics Chromium Single Cell Immune Profiling Solution kit and workflow (10x Genomics). Cells were thawed, washed twice and resuspended in RPMI containing 10% FBS to a final concentration of 100–1,000 cells per μl as determined by Cell Countess. Samples with unique donor identities were pooled together and processed for a targeted cell recovery of 10,000 cells. scRNA-seq and TCR-seq libraries were assessed for quality and quantified using the Agilent 2100 Bioanalyzer System and Qubit 3.0 Fluorometer. Single-cell RNA libraries were sequenced on an Illumina NovaSeq to a minimum sequencing depth of 25,000 reads per cell using read lengths of 26 bp read 1, 8 bp i7 index and 98 bp read 2. The single-cell TCR libraries were sequenced on an Illumina HiSeq and NovaSeq to a minimum sequencing depth of 5,000 reads per cell using read lengths of 150 bp read 1, 8 bp i7 index and 150 bp read 2. DNA was extracted from each sample donor’s CAR T cell product using the DNeasy Blood and Tissue kit (QIAGEN) and recommended protocol for Purification of Total DNA from animal blood or cells. Isolated DNA was genotyped with Infinium Omni5-4 Beadchip Array at City of Hope’s Integrative Genomics Core.

A full description of the methods and code used to process and analyze the scRNA-seq data is available at https://github.com/pachterlab/DBALLSMRDMCMGWSTPMBDKPFP_2023.

### CTC analysis

CTCs were evaluated by the Convergent Science Institute in Cancer of the USC Michelson Center for Convergent Bioscience. In short, peripheral blood and bone marrow aspirate samples were collected and shipped to the laboratory for processing and plating onto glass slides before staining with an immunofluorescence assay and imaging via automated high-throughput fluorescence scanning microscopy at ×100 magnification^11–13^^,35^. Rare events (for example, CTCs) were detected and classified using a custom computational methodology as previously reported^11–13^^,35^.

### Immunohistochemistry

IHC was performed on Ventana Discovery Ultra IHC automated stainer (Ventana Medical Systems, Roche Diagnostics) after optimization of the best-performing PSCA antibody. Formalin-fixed paraffin-embedded samples were sectioned at 5 μm and mounted on positively charged glass slides. The slides were deparaffinized, rehydrated and incubated with endogenous peroxidase activity inhibitor and antigen retrieval solution. Then the polyclonal anti-PSCA primary antibody (Abcam, cat. no. ab15168), used at 1:20 dilution, DISCOVERY Anti-Rabbit HQ and DISCOVERY Anti-HQ-HRP were incubated followed by DISCOVERY Amplification HQ kit and DISCOVERY Amplification Anti-HQ HRP multimer. The stains were visualized with a DISCOVERY ChromoMap DAB kit (Ventana) and counterstained with hematoxylin and coverslipped. The IHC stained slides were reviewed by a technical supervisor for test acceptability and pathologist for antigen sensitivity and specificity.

### Multispectral immunofluorescence tumor analysis

Formalin-fixed paraffin-embedded samples were decalcified and then cut at 4-µm sections for placement onto glass slides. Slides were baked, deparaffinized in xylene washes and rehydrated in decreasing ethanol concentration washes. Heat-induced antigen retrieval was performed using AR9 buffer, 10× (pH 9) (AR9001KT, Akoya Biosciences) in a microwave oven for 20 min. A 2-min Milli-Q water wash and TBS Automation Wash Buffer, 20× (TWB945M, Biocare Medical) wash was performed subsequently. Blocking was performed for 10 min using Antibody Diluent with Background-Reducing Components (S302283-2, Agilent) to minimize nonspecific background staining. Primary antibodies used were stained with successive rounds of secondary antibody and immunofluorescent labeling. Primary antibodies were stained in the following order: CD8 (clone 4B11, Leica CD8-4B11-L-CE), PD-L1 (clone SP142, Abcam ab228462), Ki-67 (clone SP6, Biocare CRM325B), CD3 (clone LN10, Leica NCL-L-CD3-565), Granzyme B (clone 11F1, Biocare ACI3202A) and pan-CK (clone AE1/AE3, Dako M3515). Primary antibodies were incubated for 1 h on a shaker at room temperature followed by a 10-min incubation of horseradish peroxidase (HRP)-conjugated secondary antibody (Mach 2 Rabbit or Mouse HRP-Polymer) (RHRP520 L or MHRP520 L, Biocare Medical). Immunofluorescent labeling of antibodies was achieved using the OpalTM 7-color fluorescence IHC kit (Akoya Biosciences) at a 1:100 dilution for 10 min. To perform multicolor immunofluorescent staining, the slides were serially stained with the microwave incubation acting to remove previous antibodies while simultaneously exposing the next epitope of interest. After staining the final marker, cell nuclei were stained with DAPI (FP1490, Akoya Biosciences) and the slides were mounted with ProLong Gold Antifade Reagent (P36930, Thermo Fisher Scientific). Whole-slide tissue images were acquired at ×20 using the Vectra 3.0 automated quantitative pathology imaging system (Akoya Biosciences). Multispectral images were unmixed with inForm tissue analysis software (Akoya Biosciences) and component TIFFs were exported. Using quantitative pathology and bioimage analysis (QuPath) software, the component TIFFs were stitched and examined for positive staining.

### Statistical analysis

Rates and 95% Clopper Pearson exact binomial confidence limits are presented for DLTs, PSA decrease, stable disease and 6-month survival by DL. Counts and percentages are presented for adverse events and demographics by DL. This work was conducted using R v.4.2.1 with Rstudio v.2023.6.0.421. Data are presented as mean ± s.e.m. unless otherwise stated. Statistical comparisons between groups were performed using an unpaired two-tailed Student’s *t*-test to calculate *P* values unless otherwise stated. GraphPad Prism v.8 (GraphPad Software) was used to generate bar plots and graphs.

### Obtaining biologic materials

CAR T cells were manufactured at the City of Hope in the GMP facility, with materials and processes approved by a US FDA Investigational New Drug Application. These were provided (administered) only to individual patients enrolled on the trial.

### Reporting summary

Further information on research design is available in the Nature Portfolio Reporting Summary linked to this article.

## Online content

Any methods, additional references, Nature Portfolio reporting summaries, source data, extended data, supplementary information, acknowledgements, peer review information; details of author contributions and competing interests; and statements of data and code availability are available at 10.1038/s41591-024-02979-8.

### Supplementary information

Supplementary Information Supplementary Figs. 1–4 and Tables 1–4.

Reporting Summary

### Source data

Source Data Source data for Figs. 2–4 and Extended Data Figs. 1–8.

## Figures and Tables

**Extended Data Table 1:**
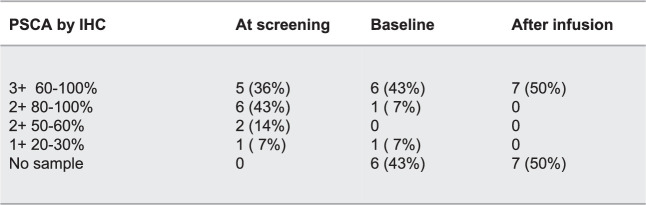
PSCA IHC scores

**Extended Data Table 2:**
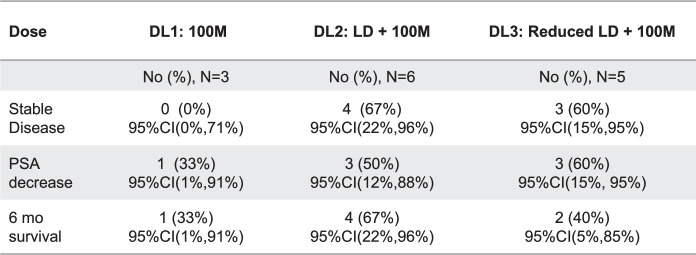
Treatment responses

## Data Availability

All required clinical data have been uploaded to ClinicalTrials.gov. All requests for raw and analyzed data and materials should be addressed to the corresponding authors and will be reviewed by the institution to verify whether the request is subject to any intellectual property or confidentiality obligations. Patient data may be subject to patient confidentiality. Any data and materials that can be shared will be released via a material transfer agreement. Source data are provided with this paper.

## Code availability

A description of the methods and the code used to process and analyze the scRNA-seq and TCR-seq data is available at https://github.com/pachterlab/DBALLSMRDMCMGWSTPMBDKPFP_2023. The TCR and single-cell RNA-seq data can be accessed at https://www.dropbox.com/scl/fo/rhgr2y28az1e2h0avaj0l/h?rlkey=6a4wlnus0png19mb80g42uja1&dl=0 (using password ‘coh_128781’).
