# Supplementary material for: PSCA-CAR T cell therapy in metastatic castration-resistant prostate cancer: a phase 1 trial
Source: Nat Med. 2024 Jun 12;30(6):1636–44. doi: 10.1038/s41591-024-02979-8 (PMC11186768; doi:10.1038/s41591-024-02979-8)
Supplement: Supplementary file 1 — Supplementary Figs. 1–4 and Tables 1–4. [file 41591_2024_2979_MOESM1_ESM.pdf]

nature medicine

Supplementary information https://doi.org/10.1038/s41591-024-02979-8

PSCA-CAR T cell therapy in metastatic castration-resistant prostate cancer: a phase 1 trial

In the format provided by the authors and unedited

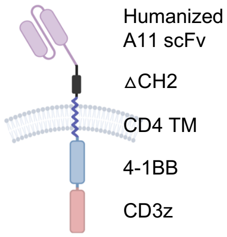

Supplementary Figure 1. PSCA-CAR construct.

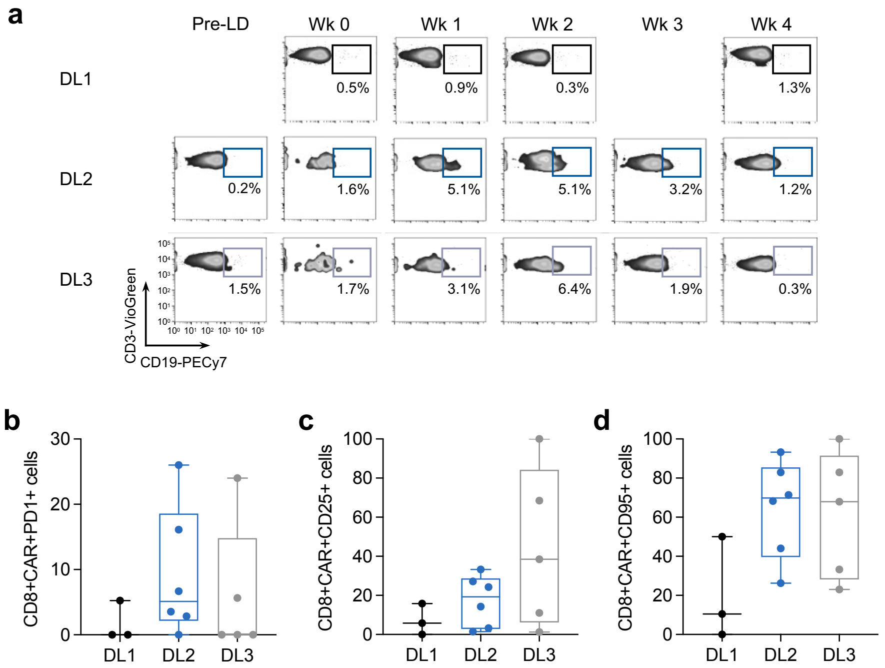

Supplementary Figure 2. Peripheral blood CAR T cell expansion and phenotype. (a) Representative patient per DL showing peripheral blood CAR T cell persistence following T cell infusion. **(b-d)** Expression of PD1 (b), CD25 (c), and CD95 (d) among CD8+CAR+ T cells in peripheral blood at peak CAR T cell expansion for each patient in each DL (DL1 n = 3, DL2 n = 6, DL3 n = 5). Data are presented as mean values ± SEM.

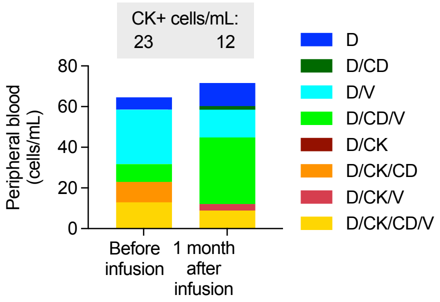

Supplementary Figure 3. Peripheral blood CTC analysis in UPN388.

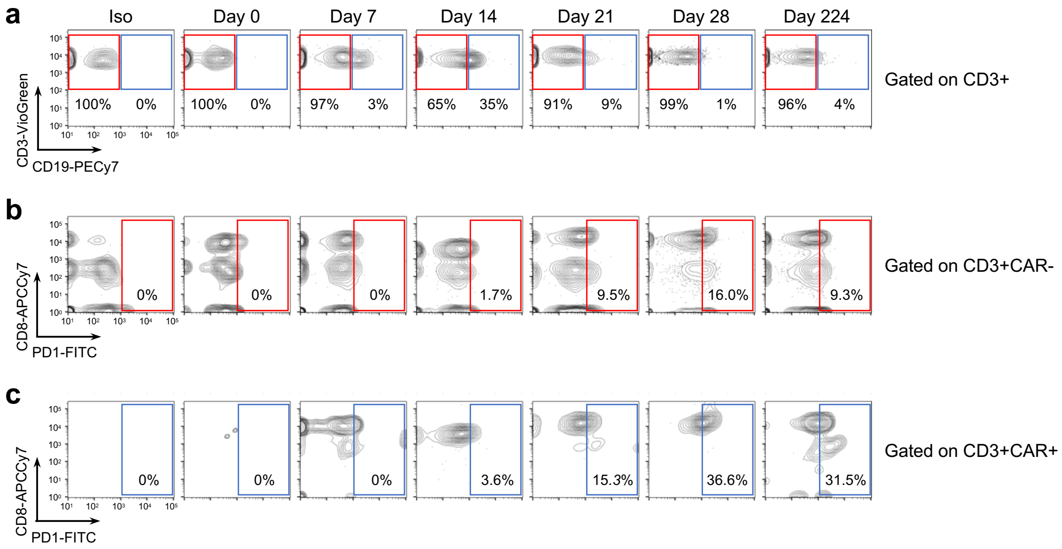

Supplementary Figure 4. Flow cytometric analysis of peripheral blood CD8+PD1+ CAR and non-CAR T cells in UPN388. (a) Flow cytometric analysis of expression of CD3+CD19+ CAR and CD3+CD19- non-CAR T cells in peripheral blood for UPN 388. **(b-c)** Expression of PD1 among CD3+CAR- non-CAR (b) and CD3+CAR+ CAR (c) T cells.

	DL1 (N=3)	DL2 (N=5)	DL3 (N=4*)
**Mean +/- STD**			
Total CK+	5.43 +/- 7.47	-12.33 +/- 19.85	-13.80 +/- 77.09
D|CK|V|CD	2.51 +/- 4.71	-6.39 +/- 19.15	-12.70 +/- 30.38
D|CK|CD	3.61 +/- 1.58	-0.81 +/- 2.90	2.04 +/- 45.52
D|CK	-0.83 +/- 3.08	-5.81 +/- 11.67	-3.21 +/- 5.92
D|CK|V	0.15 +/- 0.82	0.68 +/- 1.16	0.07 +/- 0.53
**Median**			
Total CK+	1.20	-3.34	-11.15
D|CK|V|CD	-0.82	-1.04	-1.72
D|CK|CD	3.85	-1.40	-3.56
D|CK	-0.80	-0.99	-1.82
D|CK|V	0.00	0.00	0.00

*The patient that received 2 cycles of therapy is treated as 2 unique timepoints.

Supplementary Table 1. CTC analysis in bone marrow.

Cohort	N	DLTs	CRS
DL1: 100M	3	0 (0%)<br>95%CI (0%,71%)	1 Grade 2
DL2: LD + 100M	6	2 (33%)<br>95%CI (4%,78%)	1 Grade 1<br>1 Grade 2
DL3: Modified LD + 100M	5	0 (0%)<br>95%CI (0%,52%)	2 Grade 1

Supplementary Table 2. Dose-limiting toxicities (DLTs) and cytokine release syndrome (CRS).

Marker	Fluorophore	Clone	Manufacturer	Catalog #
PD1	FITC	EH12.2H7	BioLegend	329936
CD25	APC	2A3	BD Biosciences	340939
CD137	PE	4B4-1	BD Biosciences	555956
CD19	PE-Cy7	SJ25C1	BD Biosciences	557835
CD8	APC-Cy7	SK1	BD Biosciences	348793
CD3	BV510	UCHT1	BD Biosciences	563109
CD95	PerCP-Cy5.5	DX2	BD Biosciences	561655
CD33	FITC	P67.6	BD Biosciences	340533
CD11b	APC	M1/70.15.11.5	Miltenyi Biotec Inc.	130-113-231
CD86	PE	2331 (FUN-1)	BD Pharmingen	555658
CD14	PE-Cy7	MφP9	BD Biosciences	562698
HLA-DR	APC-eFluor 780	LN3	eBioscience	47-9956-42
CD16	BV510	B73.1	BD Horizon	740203
CD15	PE-Cy5	W6D3	BioLegend	323014

Supplementary Table 3. MACSQuant 10 antibody list.

Marker	Fluorophore	Clone	Manufacturer	Catalog #
CD3	Alexa Fluor 532	UCHT1	eBioscience	58003842
CD4	PerCP	SK3	BD Biosciences	347324
CD8	BV570	RPA-T8	BioLegend	301038
CD45RA	BV650	HI100	BD Biosciences	563963
CD45	Alexa Fluor 700	HI30	BD Biosciences	560566
CD62L	BV711	SK11	BD Biosciences	565040
CD19	V450	HIB19	BD Biosciences	560353
CD39	BV510	A1	BioLegend	328220
CD69	BV750	FN50	BioLegend	310954
CD14	FITC	MphiP9	BioLegend	363508
CX3CR1	PE-Cy7	2A9-1	BioLegend	341612
LD	7AAD	-	eBioscience	00-6993-50
CXCR3	PE	G025H7	BioLegend	353706
PD-1	BV421	MIH4	BD Biosciences	BDB564323
CD28	APC-Cy7	CD28.2	BioLegend	302966
LAG-3	APC	3DS223H	eBioscience	17-2239-42
Tim3	PE-Cy5.5	F38-2E2	eBioscience	35-3109-42
CD16	BV786	3G8	BD Biosciences	563689
CCR4	Alexa Flour 647	L291H4	BioLegend	359404
CD27	PE-CF594	M-T271	BD Biosciences	BDB562297
CCR7	BV605	4B12	BioLegend	353224
CD45RO	BV480	UCHL1	BD Biosciences	BDB566143
CD25	PerCPeFluor710	BC96	eBioscience	46-0259-42
PD-L1	PE-Fire810	29E.2A3	BioLegend	329755
CD95	APC-Fire 810	DX2	BioLegend	305663

Supplementary Table 4. Cytek Aurora 3 antibody list.
