# Supplementary material for: PSCA-CAR T cell therapy in metastatic castration-resistant prostate cancer: a phase 1 trial
Source: Nat Med. 2024 Jun 12;30(6):1636–44. doi: 10.1038/s41591-024-02979-8 (PMC11186768; doi:10.1038/s41591-024-02979-8)
Supplement: Supplementary file 2 — Reporting Summary [file 41591_2024_2979_MOESM2_ESM.pdf]

nature portfolio

Corresponding author(s): Dorff TB

Last updated by author(s): 2024 Apr 3

Reporting Summary

Nature Portfolio wishes to improve the reproducibility of the work that we publish. This form provides structure for consistency and transparency in reporting. For further information on Nature Portfolio policies, see our Editorial Policies and the Editorial Policy Checklist.

Statistics

For all statistical analyses, confirm that the following items are present in the figure legend, table legend, main text, or Methods section.

n/a	Confirmed	
☐	☒	The exact sample size (*n*) for each experimental group/condition, given as a discrete number and unit of measurement
☐	☒	A statement on whether measurements were taken from distinct samples or whether the same sample was measured repeatedly
☐	☒	The statistical test(s) used AND whether they are one- or two-sided<br>*Only common tests should be described solely by name; describe more complex techniques in the Methods section.*
☐	☒	A description of all covariates tested
☒	☐	A description of any assumptions or corrections, such as tests of normality and adjustment for multiple comparisons
☐	☒	A full description of the statistical parameters including central tendency (e.g. means) or other basic estimates (e.g. regression coefficient) AND variation (e.g. standard deviation) or associated estimates of uncertainty (e.g. confidence intervals)
☒	☐	For null hypothesis testing, the test statistic (e.g. *F*, *t*, *r*) with confidence intervals, effect sizes, degrees of freedom and *P* value noted<br>*Give P values as exact values whenever suitable.*
☒	☐	For Bayesian analysis, information on the choice of priors and Markov chain Monte Carlo settings
☒	☐	For hierarchical and complex designs, identification of the appropriate level for tests and full reporting of outcomes
☒	☐	Estimates of effect sizes (e.g. Cohen's *d*, Pearson's *r*), indicating how they were calculated

Our web collection on statistics for biologists contains articles on many of the points above.

Software and code

Policy information about availability of computer code

Data collection	Trial data was collected using Medidata RAVE®; COH's electronic data capture (EDC) system. Note: RAVE is a web-based, password protected application that is fully compliant with global regulatory requirements, including 21CRF Part 11.
Data analysis	Data analysis was done using R 4.2.3

For manuscripts utilizing custom algorithms or software that are central to the research but not yet described in published literature, software must be made available to editors and reviewers. We strongly encourage code deposition in a community repository (e.g. GitHub). See the Nature Portfolio guidelines for submitting code & software for further information.

Data

Policy information about availability of data

All manuscripts must include a data availability statement. This statement should provide the following information, where applicable:

- Accession codes, unique identifiers, or web links for publicly available datasets
- A description of any restrictions on data availability
- For clinical datasets or third party data, please ensure that the statement adheres to our policy

All required clinical data have been uploaded to clinicaltrials.gov
TCR and scRNAseq data will be uploaded to GitHub

Human research participants

Policy information about studies involving human research participants and Sex and Gender in Research.

Reporting on sex and gender	Because prostate cancer only affects people with a prostate (genetic males) there was no sex/gender analysis in this phase 1 study involving only prostate cancer.
Population characteristics	Population characteristics are summarized in the manuscript Table 1
Recruitment	Patient were recruited from the patient population, existing and referred, to City of Hope in Duarte CA. This may have resulted in bias for patients who were not physically or financially able to travel, though participants from other states did end up enrolling, and travel support was sought for participants coming from a distance, suggesting some of this bias may have been less severe than it could have been.
Ethics oversight	City of Hope IRB approved and oversaw the conduct of the study

Note that full information on the approval of the study protocol must also be provided in the manuscript.

Field-specific reporting

Please select the one below that is the best fit for your research. If you are not sure, read the appropriate sections before making your selection.

☒ Life sciences ☐ Behavioural & social sciences ☐ Ecological, evolutionary & environmental sciences

For a reference copy of the document with all sections, see nature.com/documents/nr-reporting-summary-flat.pdf

Life sciences study design

All studies must disclose on these points even when the disclosure is negative.

Sample size	the TEQR design of Blanchard and Longmate was used for planning dose escalation. The sample size could have been larger, but ethically we felt that further dose escalation was not appropriate based on the toxicity and efficacy seen in the patients who were treated. The sample size is adequate to have determined the DLT and toxicity profile of PSCA CAR T cells and to plan a phase 1b dosing strategy aimed at enhancing benefit:risk ratio for subsequent participants.
Data exclusions	No data were excluded
Replication	Human subjects represent essentially an "n of 1" and each subjet's experience cannot be replicated. however the TEQR design intends to contain enough different subjects to capture adequate experiences to determine DLT.
Randomization	n/a - no randomization
Blinding	n/a - no blinding

Reporting for specific materials, systems and methods

We require information from authors about some types of materials, experimental systems and methods used in many studies. Here, indicate whether each material, system or method listed is relevant to your study. If you are not sure if a list item applies to your research, read the appropriate section before selecting a response.

Materials & experimental systems

n/a	Involved in the study	
☒	☐	Antibodies
☒	☐	Eukaryotic cell lines
☒	☐	Palaeontology and archaeology
☒	☐	Animals and other organisms
☐	☒	Clinical data
☒	☐	Dual use research of concern

Methods

n/a	Involved in the study	
☒	☐	ChIP-seq
☐	☒	Flow cytometry
☒	☐	MRI-based neuroimaging

Clinical data

Policy information about clinical studies

All manuscripts should comply with the ICMJE guidelines for publication of clinical research and a completed CONSORT checklist must be included with all submissions.

Clinical trial registration	NCT03873805
Study protocol	Study protocol is attached as appendix
Data collection	Subjects were recruited between 2018 and 2022 at City of Hope in Duarte, CA. all data were collected at City of Hope
Outcomes	Primary outcome was dose limiting toxicity, and was evaluated based on CTC AE toxicities meeting certain grades as defined in the protocol.<br>Secondary outcomes included CAR T expansion, proliferation, cytokine induciton, and anti-tumor effect measured as PSA and radiographic responses.

Flow Cytometry

Plots

Confirm that:

- [x] The axis labels state the marker and fluorochrome used (e.g. CD4-FITC).
- [x] The axis scales are clearly visible. Include numbers along axes only for bottom left plot of group (a 'group' is an analysis of identical markers).
- [x] All plots are contour plots with outliers or pseudocolor plots.
- [x] A numerical value for number of cells or percentage (with statistics) is provided.

Methodology

Sample preparation	Peripheral blood samples were obtained from subjects prior to and at various timepoints for 28 days following CAR T cell infusion, as well as day 60, 90, and q12 weeks after day 90 to evaluate CAR T cell expansion/persistence. Peripheral blood samples were lysed using BD PharmLyse (15 min at RT) and quenched using RPMI containing 10% FBS. Cells were resuspended in FACS buffer (Hank's balanced salt solution without Ca2+, Mg2+, or phenol red (HBSS−/−, Life Technologies) containing 2% FBS and 1 × AA). Cells were incubated with Fc block (BD Biosciences) for 5 min at RT and then incubated with fluorescence-labelled antibodies for 15 min at RT in the dark. Unless otherwise stated, antibodies were used at a dilution of 1:100
Instrument	MACSQuant Analyzer 10 (Miltenyi Biotec) or Cytek Aurora 3
Software	FlowJo software (v10.8.1, TreeStar) or OMIQ software (Dotmatics)
Cell population abundance	When possible, at least 10,000 events were collected for each flow cytometry sample. In some cases, as few as 50 cells were gated to detect low populations of CAR+ T cells
Gating strategy	Relevant populations were gated as follows: Live cells --> gating of interest (i.e. CD3+ for T cells and CD3+CD19+ for CAR+ T cells). Negative and positive controls were included in each assay, when available. Refer to Figure S10 for flow cytometry representative gating strategy.

- [x] Tick this box to confirm that a figure exemplifying the gating strategy is provided in the Supplementary Information.
